# Microwave Metamaterial-Based Sensor for Dielectric Characterization of Liquids

**DOI:** 10.3390/s18051513

**Published:** 2018-05-11

**Authors:** André Soffiatti, Yuri Max, Sandro G. Silva, Laércio M. de Mendonça

**Affiliations:** Department of Communication Engineering, Federal University of Rio Grande do Norte, Campus Universitário, Natal RN 59078-900, Brazil; yuri@gmail.com (Y.M.); sandro@ct.ufrn.br (S.G.S.); laercio@ct.ufrn.br (L.M.d.M.)

**Keywords:** microstrip sensor, metamaterial, microstrip antenna, quality control, measurement method

## Abstract

This article proposed to build a system founded on metamaterial sensor antennas, which can be used to evaluate impurities in aqueous substances according to the quality of transmission between the sensor antennas. In order to do this, a dedicated setup with tests in several frequencies was deployed so as to monitor the behavior of transmission variation between sensors. These sensors are microstrip antennas with a ground plane of resonant cleaved metallic rings; the substrate functions as a metamaterial for the irradiating element. In this study, an analysis was made of transmission between the sensors, looking for variation in angles of incidence of signal and of distance between the antennas. The sensor was tested at various operating frequencies, as such 1.8 GHz, 2.4 GHz, 3.4 GHz and 4.1 GHz, resulting in different values of sensitivity. The prototypes were constructed and tested so as to analyze the dielectric effects of the impurities on NaCl and C_2_H_4_O_2_ substances. The research aims to use these control systems of impurities in industrial premises.

## 1. Introduction

Recently, the development of sensor technology has been evident in different applications, such as automobiles, aircraft, and industries and even in residences.

The common approach to measure the dielectric properties of solid, liquid, and gaseous materials is to quantify their interaction within the fields of an electromagnetic wave [1]. This provides a contactless measurement of electrical properties such as permittivity and conductivity [2]. For liquids, which are the focus of this paper, there is a wide spectrum of information—such as relaxation processes and orientation polarization of the molecules—all of which contribute to the liquid’s complex dielectric function and its frequency and temperature variations.

Microwave approaches (1 GHz–10 GHz) offer means of liquid characterization. The polar nature of the liquids makes their molecules interact with the electric field of electromagnetic waves over microwave frequencies. Therefore, it should be promising to design and realize microwave sensors which utilize this interaction for liquid characterization. The accuracy of dielectric measurement of liquids is still challenging in many applications. Currently, although there are many types of microwave sensors, it is known that there are still problems to be solved. The problems addressed can be summarized as: accuracy, size, cost, simplicity, real-time measurements, and electromagnetic waves transmission efficiency.

Previous studies investigated a highly sensitive microwave-coupled ring resonator with a wide dynamic range, studied for its use in sensing applications [3]. One study [4] demonstrated an improved electromagnetic energy without volume detection. The microwave microfluidic sensor based on microstrip-line-coupled complementary resonator was analyzed [5]. The calculation the complex permittivity of the solution and the measurements are used for the verification of the sensing principle [6]. The sensor has been presented as an active feedback loop-assisted planar microwave resonator operating at 1.5 GHz [7]. An innovating flow sensor is used to measure the flow rate within microchannels in a real-time, noncontact and nonintrusive manner. In this case, the microfluidic device is made of a fluidic microchannel sealed by a thin polymer layer interfacing the fluidics and microwave electronics [8]. Another approach was developed by Abdolrazzaghi et al. [9], whose objective was to develop a novel wireless high-resolution resonant-based microwave sensor, to be used in chemical-sensing applications.

This paper presents an experimental technique to evaluate the degree of impurity in liquid substances (microfluidics) based on their complex dielectric permissiveness using sensor antennas with metamaterials, to thus obtain a standard by which to control for the variation of the intrinsic characteristics of these substances [10,11,12].

This article proposes the study and development of new sensors with metamaterials. The technique consists of analyzing the transmission of signals between sensor antennas. In this way, information can be obtained by concentrating different aqueous substances to define the complex permissiveness of the liquid. A pair of microstrip antennas with a metamaterial substrate was used; microstrip antennas are especially attractive due to their low cost, low profile and easy integration with other circuits and elements [13].

The rings deploying metamaterial substrate are artificially manufactured electromagnetic materials, consisting periodically of metallic elements that are smaller than the wavelength of the incidental electromagnetic wave (EM) in size. In addition, metamaterials manipulate electromagnetic wave beams in surprising ways, and exhibit some different electromagnetic properties that are not readily available in nature [14,15,16,17,18]. Metamaterials are plasmonics-based devices that have significant applications in chemical and biomedical sensing, surface enhanced spectroscopy, and near-field scanning optical microscopy. The excitations of metamaterial and plasmonic structures lead to localized electric and magnetic resonances which provide excellent electromagnetic sensing [19]. In recent years they have attracted considerable attention because of their negative refractive index [20,21] and grow thin the high frequency of gain and bandwidth [22,23,24].

In this work, a model was developed from an Ultra-Wideband (UWB) microstrip resonator antenna, with the substrate modified by metallic rings to allow changing magnetic permeability. The idea was to investigate the maximum energy transfer between sensor antennas.

The research development consisted of theoretical studies, computational simulations and measurements. In the methodological procedures the modified substrate was developed so that it approximates its intrinsic impedance with the external environment impedance and reduces the reflection coefficient in the antenna. In this method is used the most of the energy supplied by the signal source in the irradiation process [25,26,27,28].

It is hoped that this research will help other researchers through providing a deeper perspective on metamaterial sensor antennas, with the hope that it can be considered as an alternative that produces a better technical design.

## 2. Materials and Methods

### 2.1. Metamaterial Sensor

Broadband antenna designs are widely used in many applications. Moreover, in this study the environment is usually controlled due to the tradeoff between gain and directivity of bandwidth [29]. As a method of detection of the sensor, the parameters of permissiveness, electrical and magnetic permeability were used in order to allow the choice of parameters in a frequency range. The sensors are broadband antennas used for the proposed sensitivity method test. The transmitting antenna (Tx) sends a signal through an aqueous substance to the receiving antenna (Rx). The aqueous substances are test samples which have their compositions changed to modify their electrical properties, since an electromagnetic wave depends on the relative permittivity and relative permeability of the medium. Thus, the transmission suffers different effects due to impurities in the sample which are not felt in the transmission module. Several frequencies were tested to verify the sensitivity variance of the sensors. From this data, the dispersion models recover physically accurate constitutive parameters throughout the analyzed frequency region.

**Figure 1:**
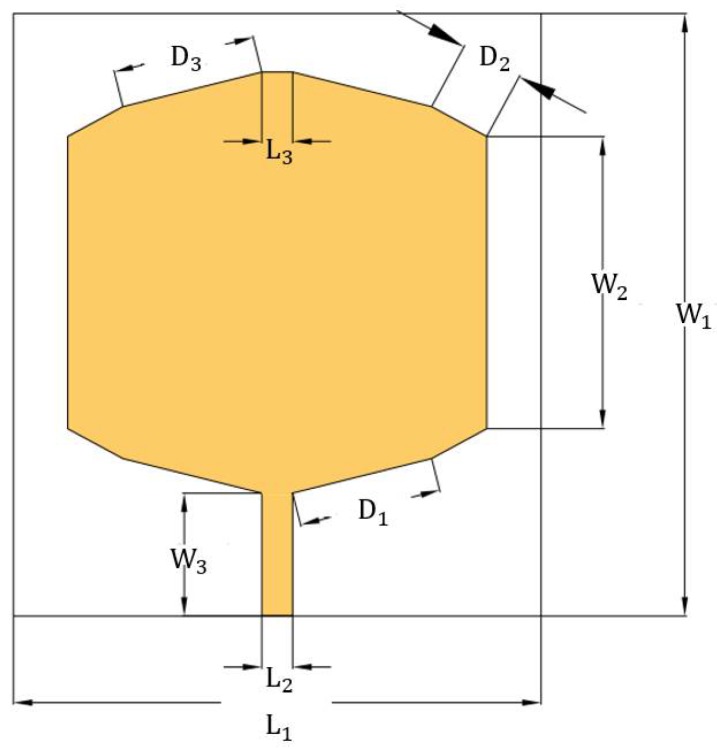
Draft of the projected antenna.

The optimized and built antenna is shown in Figure 1. Table 1 depicts the values of the dimensions of the parameters in millimeters.

**Figure 2:**
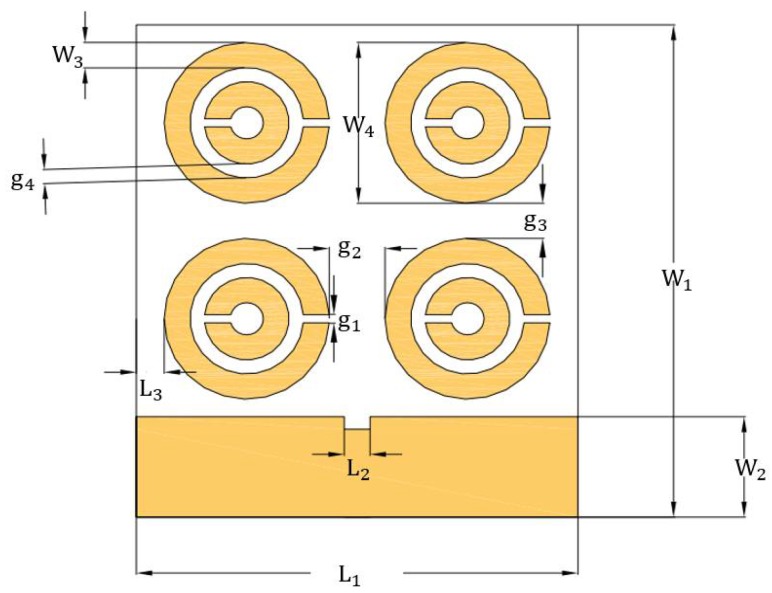
Schematic metamaterial design.

In the substrate specification, predicted changes were made to the metamaterial. Figure 2 depicts the substrate metamaterial, which is the association of polytetrafluoroethylene made of concentrated circular metal rings constructed on the opposite face to the element radiator [30,31,32].

In Table 2, the parameters of the metamaterial are shown.

The substrate material used in the sensors is a low-cost glass fiber with a tangent loss of 0.01. The losses of the interaction of the wave with the substrate made as the wave passes between the ground plane, the metamaterial reflectors and the irradiator element are not significant to transmission results of the sensor sensitivity method, since only the final transmission is analyzed in the sensitivity curves.

For our experiment, we will control the transmission environment, so that the antenna weaknesses will not be significant.

**Figure 3:**
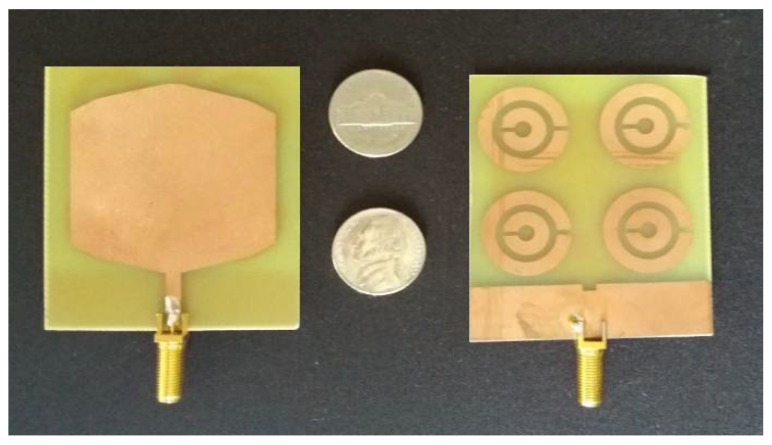
Sensor photography.

The physical construction shown in Figure 3 was made by the corrosion. The final design was simulated in the High Frequency Structure Simulator (HFSS) with the appropriate fine-tune adjustments. The result of this is shown below, compared with dollar coins for scale.

All calculations were coded in MATLAB, using the substrate glass fiber with εr = 4.4, t = 0.05 mm, h = 1.60 mm. The simulation was done in the HFSS [33], obtaining the data described in Figure 1.

**Figure 4:**
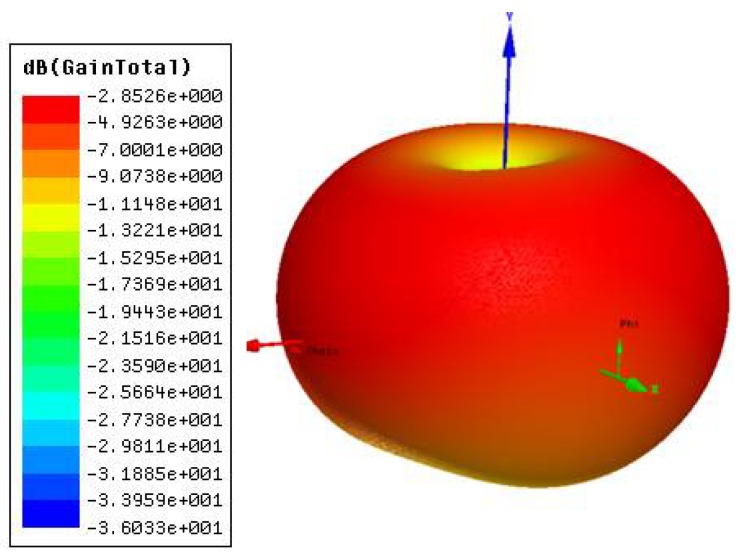
Radiation diagram.

The 3D radiation of the projected antenna in Figure 4 is shaped like an apple, which concentrates the highest gain near the YZ plane. The antenna is placed in the XY plane, with power coming from the Y axis to feed the antenna.

**Figure 5:**
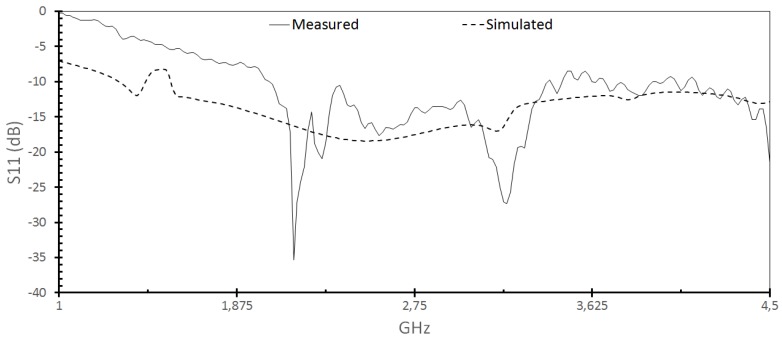
Return loss S11 (dB) versus frequency (GHz) for the sensor antennas.

The simulated parameter S_11_ of the antenna in Figure 5 demonstrates its high capacity to operate at several frequencies as a UWB antenna. Using a criterion of −10 dB, bandwidth starts at 1 GHz and extends beyond 14 GHz with good impedance matching using a high loss and low-quality material, the by epoxy resins. The prototypes showed a satisfactory correlation with the simulated results Figure 3.

With the ground plane using simple metamaterial (horizontal), there is a decrease in the relative permissiveness. This helps create a patch with greater coverage on the substrate, resulting in a greater bandwidth and gain compatible with UWB. The form of the radiation and values can be seen in Figure 4. The S_11_ parameter of the sensors did not result in a significant change in their response with the presence of water samples with NaCl and C_2_H_4_O_2_ concentrations, because the samples were too small and were not big enough to change the resonances of the antennas.

**Figure 6:**
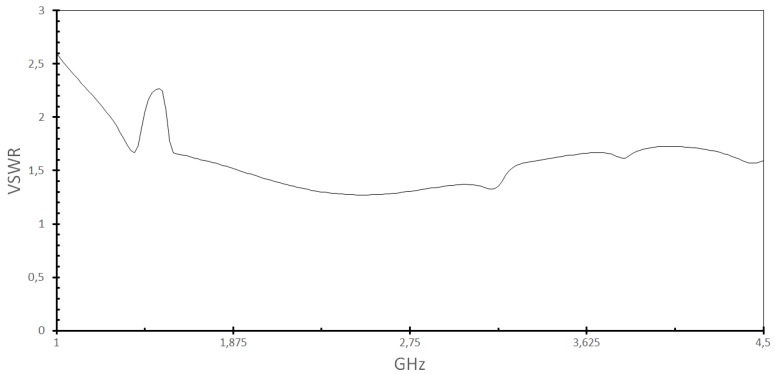
The voltage standing wave ratio (VSWR) versus frequency for the sensor antennas.

We can see the VSWR (Voltage Standing Wave Ratio) in Figure 6 of the proposed sensor. As values approximate 1, the better the antenna matches with the observed frequency, since it is the magnitude ratio module between the standing wave at a maximum point and the next minimum point. As expected, the VSWR perfectly matches the S_11_ variations of the sensor in Figure 5.

**Figure 7:**
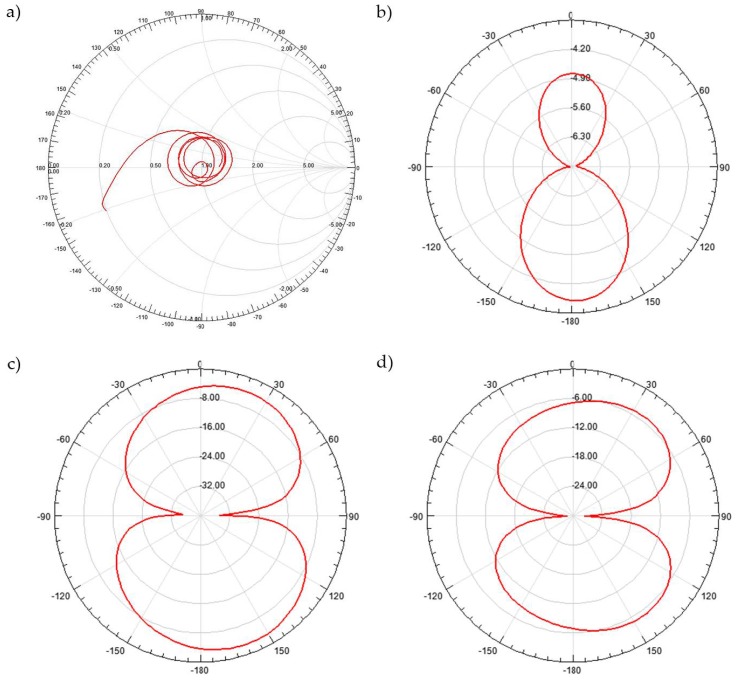
(**a**) Smith chart and gain view for (**b**) ϕ=0°; (**c**) ϕ=90° and (**d**) θ=90°.

The Smith chart shown in Figure 7a reveals the matching at each frequency; it can be seen if the structure is tending to capacitive or inductive impedances. Even in extreme cases, corrective solutions can be adopted when the result is unsatisfactory.

The comparison of gain in different observation planes is shown in Figure 7. In Figure 7b, we can see the 2D diagram of the radiation of Figure 3, when ϕ=0°, likewise, Figure 7c corresponds to the radiation when ϕ=90°, and when observing the radiation in the plane θ=90°, Figure 7d is obtained.

### 2.2. Measurement Setup

**Figure 8:**
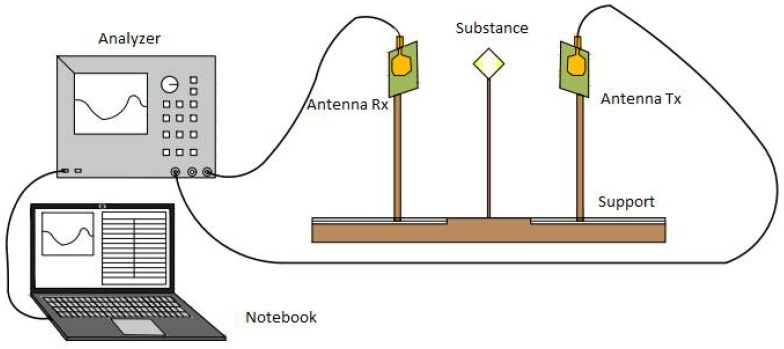
Schematic of setup of measurement.

**Figure 9:**
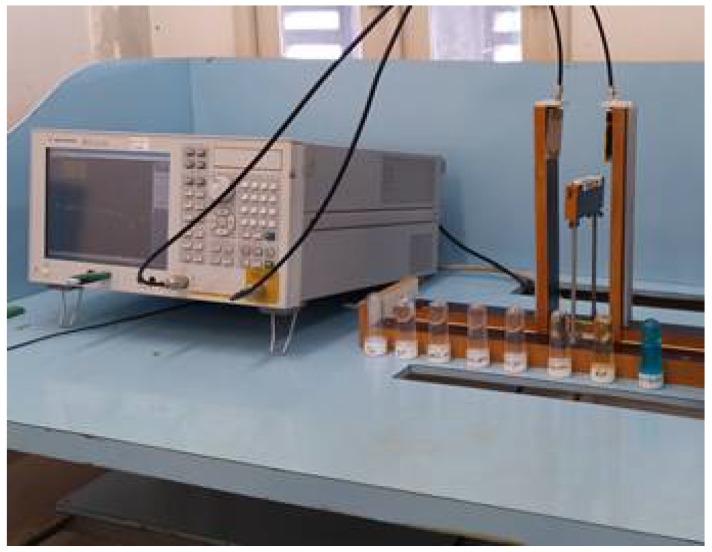
Photograph of the setup of the measuring experiment.

The measurement setup was performed within a controlled distance between the sensors, seen the project schematic shown in Figure 8 and its deployment in Figure 9. For the tests performed, the Agilent E5071C ENA Network Analyzer was used to provide new speed standards, accuracy and versatility for networks. The range of frequency varied between 1.8 GHz and 4.5 GHz.

**Figure 10:**
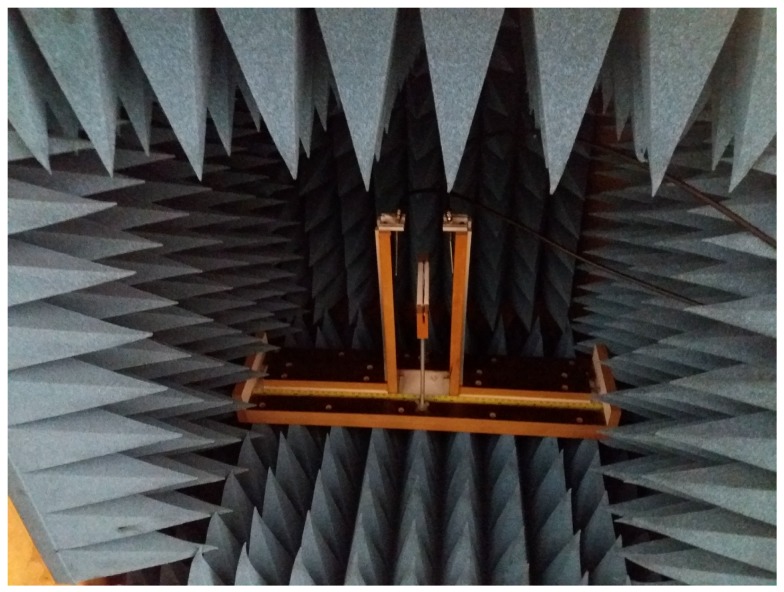
Photography of the anechoic chamber.

The anechoic chamber in Figure 10 is shown with dimensions that are most efficient against reflections and interferences from the sensor’s own radiation, which also isolate external sources of noise.

A notebook computer coupled with the system reproduces graphs, makes collecting tables, and registers the frequency range used. The transmitting and receiving sensors are microstrip antennas with circular split rings that work together with the metamaterial to obtain greater bandwidth.

### 2.3. Test Material

The samples used in the experiments were as follows: Sodium chloride (NaCl) with molar mass of 58.44 g/mol and fusion point 801 °C, classified as salt. When dissolved in 100 mL of water The sample multiples were percentages of 1%, 3%, 10%, 20% and 30%, respectively. 

The other sample that was used in the experiments was ethanoic acid (‎$C_{2}H_{4}O_{2})$ with molar mass of 60 g/mol and fusion point 6.5 °C, soluble in 100 mL of water in percentages of 1%, 3%,10%, 20%, and 30%.

The set proved to be efficient over transmission tests without high interference by the medium, which resulted in a good analysis of the operational sensors with predictive results.

## 3. Results and Discussion

The results of the tests carried out included a measurement system composed by an analyzer and microstrip antennas with metamaterial substrate in split rings, whose main purpose was to increase the frequency bandwidth for the measurement of NaCl solutions in five levels of concentration. The initial test was performed through the anechoic chamber to measure the degree of impurity at different concentrations of 1%, 3%, 10%, 20% and 30% of NaCl, which were diluted in 100 mL of water. The same method was applied to $C_{2}H_{4}O_{2}$.

In order to verify the different behaviors of the sensors, two distances and two degrees of position were used. Initially, the tests were performed within a distance of 10 cm and the angle of incidence of the signal was 90°, following that, the same tests were performed over a distance of 20 cm and angle between the sensors of 45 degrees. In the laboratory where the tests occurred the temperature was 24 °C, with a pressure of 1009 hPa and humidity of 80%. The effects of humidity over this test setup is dispensable, as the distance between the sensors is considerably small. However, it is known that the high incidence of humidity as a natural effect (rain) causes more loss of power in the signal when it propagates over a charged environment.

The analyzer stimulates the antenna in transmissions with frequencies ranging from 1.8 GHz to 4.2 GHz by generating an electromagnetic wave that is absorbed by the antenna measuring the quality of the transmission signal between the two sensors, which depends on different levels of impurity. This electromagnetic wave is transmitted to the antenna through a coaxial cable. Thus, the parameter S21 is obtained. The incidence of the electromagnetic wave on both antennas is identified, and then observed a behavior change in attenuation (dB), distinguishing each concentration used.

We can verify in Table 3, Table 4, Table 5 and Table 6 that when the frequency changed and concentration of impurities increased, a linear change occurred in the transmission behavior. With this linearity, a sensor was designed to predict in advance the change in the degree of purity of the substances. Even over frequency variation, the linear behavior was consistent.

The results of Table 3, Table 4, Table 5 and Table 6 indicate that it is possible to use electromagnetic waves sensors to monitor the concentration or impurities of previously analyzed samples, allowing the creation of low implementation cost, quality control sensors with high efficiency.

The setup proved to be very efficient for the research goals, since it guaranteed good isolation of the system, allowing configuration to fit exactly the intended experiment, allowed by the support specially developed for this experiment and the anechoic chamber.

Positioning the substances between the receiver and the transmitter enabled us to evaluate the permissiveness—and thus obtain a standard to control the variation of the intrinsic characteristics—of these substances [34]. Using a frequency range of 1.8 GHz to 4.2 GHz, the radiation generated from the sensor was directed to an aqueous solution and the results were generated in terms of the transmission quality between the sensors [35]. Depending on the chosen observed frequency there was a different sensitivity line that changed according to the degree of concentration of the substance and/or the degree of the tests performed with the sensors positioned at two different angles. This was initially at 90° and 45°, using a distance between the sensors of 10 cm from the angle of 90° and of 20 cm from the angle of 45°.

**Figure 11:**
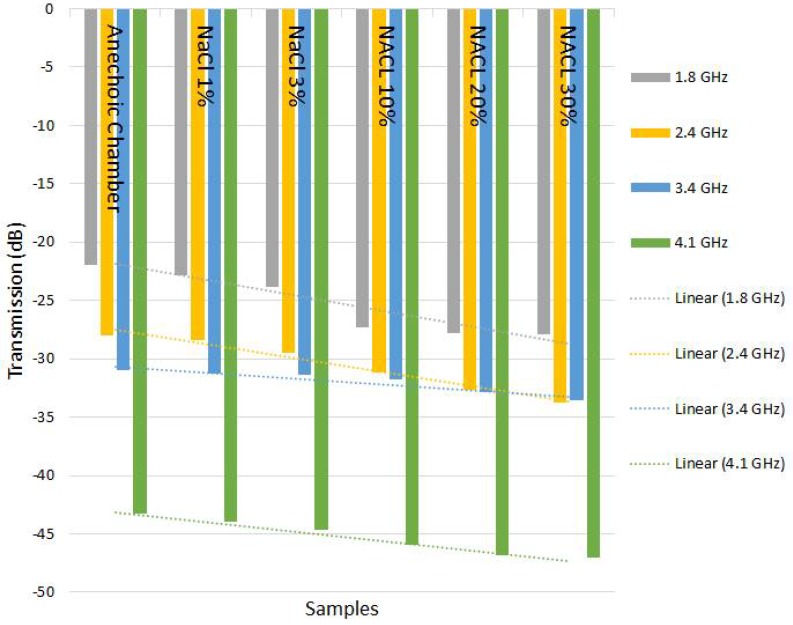
Graphs showing linear behavior in relation to frequency and type of material in the test with NaCl–angle 90° and distance 10 cm.

**Figure 12:**
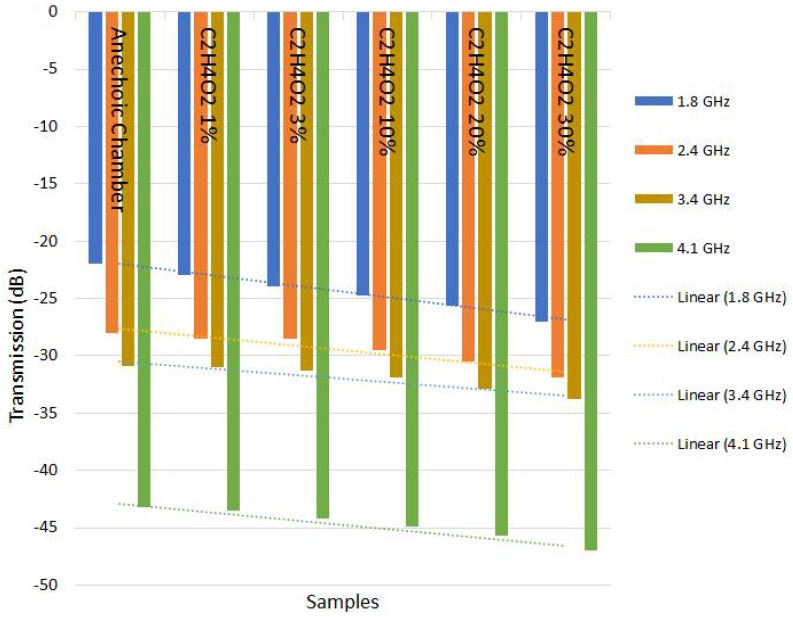
Graphs showing linear behavior in relation to frequency and type of material in the test with $C_{2}H_{4}O_{2}$–angle 90° and distance 10 cm.

**Figure 13:**
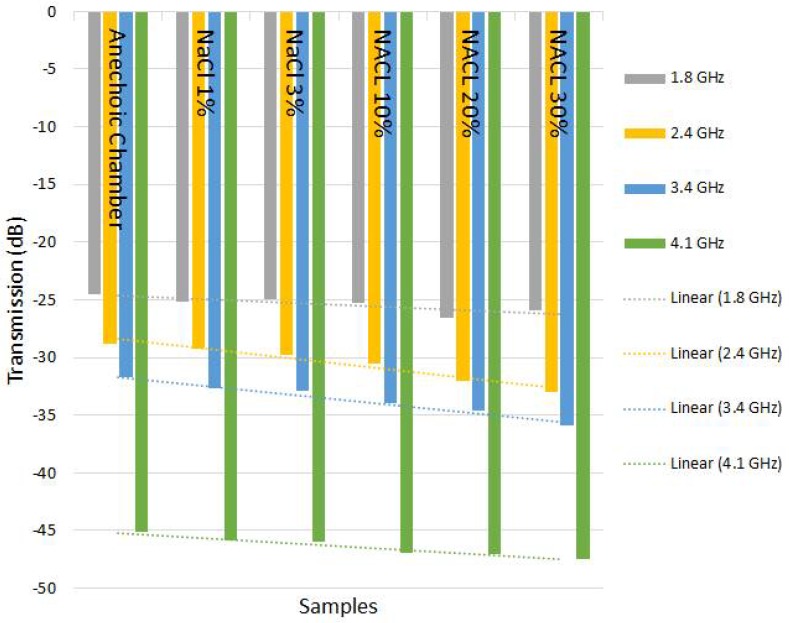
Graphs showing linear behaviors in relation to frequency and type of material in the test with NaCl–angle 45° and distance 20 cm.

**Figure 14:**
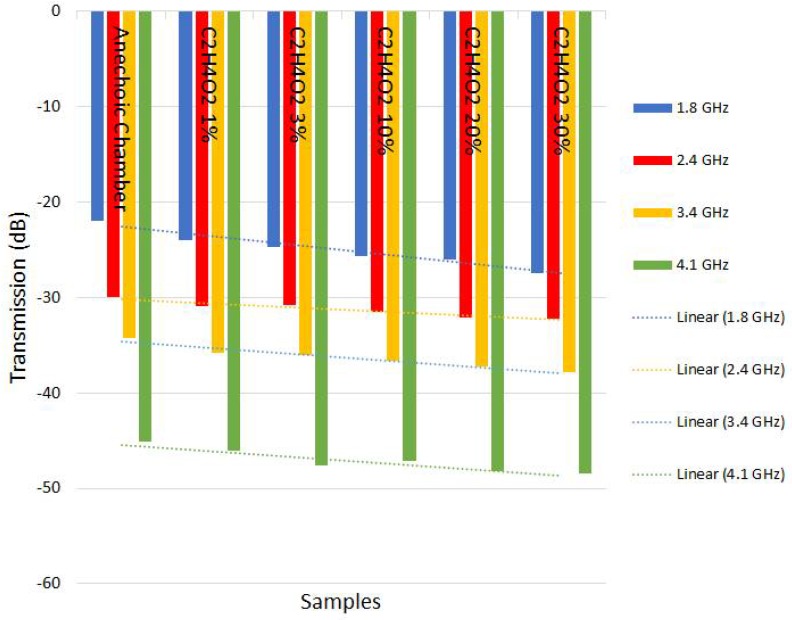
Graphs showing linear behaviors in relation to frequency and type of material in the test with $C_{2}H_{4}O_{2}$–angle 45° and distance 20 cm.

It is evident from Figure 11, Figure 12, Figure 13 and Figure 14 that even in the analyzed frequency, sensors responded in a similar way, indicating the possibility of using different frequencies to verify the purity of the substance under analysis.

Due to the behavior of attenuation, it should be possible to equate and relate the degree of impurity with the loss of transmission quality, leading to many other possible deployments for this sensor in cases where specific goals must be achieved [36].

The metamaterial microstrip antennas were chosen because of their high bandwidth despite their low efficiency, as this allowed the effect of different concentrations of impurities in an extensor frequency range to be tested. Notwithstanding the implementation of sensors for industry purposes, like the antennas with a response dedicated to operational frequency can be used after an analysis is made for the sake of cataloguing transmission behavior with different concentrations of impurities of the chosen sensor. 

Research should continue to test materials in different physical states to assess technology thresholds used to assist with quality control, and even optimize rollouts when there are materials that potentiate transmission.

The sensitivity continues with a curve equivalent to that shown in Figure 11 and Figure 12, only the transmission is affected due to distance, as expected, because power decays with the square of distance.

Using different angles for the sensors, there is a notorious difference in reception; the radiation of the antenna is not homogeneous in all directions. Use at angles other than the maximum gain of the antenna will result in negative impacts on transmission, as shown in the Figure 13 and Figure 14; however the sensitivity curves continue to be congruent. Sensors should be used at the maximum radiation angle for best utilization. With misalignment between sensors, there were significant losses in the transmission, however, it is expected that due to the increase in the distance between the antennas, a similar effect was caused when there was an increase in the distance between the aligned sensors [37].

**Figure 15:**
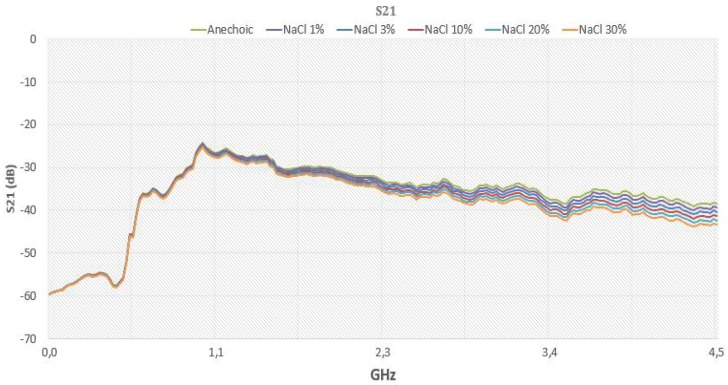
The evolution of transmission according to NaCl concentration.

Figure 15 is a comparison of the NaCl substance with the graphs, for the purpose of illustrating the behavior of the evolution of the concentration in the transmission, according to the frequency variation.

## 4. Conclusions

The proposed system showed that metamaterial microstrip antennas can be used as a substance sensor. The study involved the implementation of a sensor with good sensitivity and precision which can be used to analyze and detect a great variety in materials such as solids, liquids or mixed-materials. This is due to its capability in qualifying and characterizing the composition of a sample. The proposed sensor and associated techniques are very promising; particularly in tests carried out on liquid substances, it demonstrated good precision in the analysis of impurity of these materials. The results were consistent throughout the frequencies analyzed, allowing the chosen frequencies that did not interfere with the operation of any other devices. The research reached its initial goal and also opened a number of new possibilities for efficient and low-cost sensor sued in controlling impurities.

## Figures and Tables

**Table 1:** Antenna design parameters.

Design Parameter	Dimension (mm)	Design Parameter	Dimension (mm)
$L_{1}$	55.64	$W_{3}$	13.00
$L_{2}$	3.25	$D_{1}$	15.10
$L_{3}$	3.25	$D_{2}$	6.62
$W_{1}$	63.70	$D_{3}$	15.10
$W_{2}$	30.89		

**Table 2:** Design parameters of the metamaterial.

Design Parameter	Dimension (mm)	Design Parameter	Dimension (mm)
$L_{1}$	55.64	$W_{4}$	20.79
$L_{2}$	3.25	$g_{1}$	1.04
$L_{3}$	3.51	$g_{2}$	7.03
$W_{1}$	63.70	$g_{3}$	4.56
$W_{2}$	13.00	$g_{4}$	1.81
$W_{3}$	3.30		

**Table 3:** Transmission measured according to the frequency at different concentrations of NaCl–angle of 90° and distance of 10 cm.

Substances	S21 to 1.8 GHz	S21 to 2.4 GHz	S21 to 3.4 GHz	S21 to 4.1 GHz
Anechoic Chamber	−22.002 dB	−27.987 dB	−30.932 dB	−43.223 dB
NaCl 1%	−22.883 dB	−28.398 dB	−31.288 dB	−43.974 dB
NaCl 3%	−23.871 dB	−29.533 dB	−31.399 dB	−44.682 dB
NaCl 10%	−27.311 dB	−31.174 dB	−31.789 dB	−45.910 dB
NaCl 20%	−27.790 dB	−32.667 dB	−32.890 dB	−46.871 dB
NaCl 30%	−27.893 dB	−33.765 dB	−33.541 dB	−47.033 dB

**Table 4:** Transmission measured according to the frequency at different concentrations of $C_{2}H_{4}O_{2}$ –angle for 90° and distance 10 cm.

Substances	S21 to 1.8 GHz	S21 to 2.4 GHz	S21 to 3.4 GHz	S21 to 4.1 GHz
Anechoic Chamber	−22.002 dB	−27.987 dB	−30.932dB	−43.223 dB
C_2_H_4_O_2_ 1%	−22.992 dB	−28.506 dB	−30.994 dB	−43.547 dB
C_2_H_4_O_2_ 3%	−23.956 dB	−28.543 dB	−31.265 dB	−44.236 dB
C_2_H_4_O_2_ 10%	−24.760 dB	−29.541 dB	−31.909 dB	−44.917 dB
C_2_H_4_O_2_ 20%	−25.652 dB	−30.454 dB	−32.921 dB	−45.707 dB
C_2_H_4_O_2_ 30%	−26.994 dB	−31.867 dB	−33.764 dB	−46.986 dB

**Table 5:** Transmission measured according to the frequency at different concentrations of NaCl–angle of 45° and distance of 20 cm.

Substances	S21 to 1.8 GHz	S21 to 2.4 GHz	S21 to 3.4 GHz	S21 to 4.1 GHz
Anechoic Chamber	−24.566 dB	−28.782 dB	−31.721 dB	−45.122 dB
NaCl 1%	−25.145 dB	−29.294 dB	−32.691 dB	−45.909 dB
NaCl 3%	−24.995 dB	−29.822 dB	−32.904 dB	−46.012 dB
NaCl 10%	−25.298 dB	−30.567 dB	−33.998 dB	−46.910 dB
NaCl 20%	−26.590 dB	−31.999 dB	−34.556 dB	−46.996 dB
NaCl 30%	−25.884 dB	−33.002 dB	−35.886 dB	−47.445 dB

**Table 6:** Transmission measured according to the frequency at different concentrations of $C_{2}H_{4}O_{2}$ –angle for 45° and distance 20 cm.

Substances	S21 to 1.8 GHz	S21 to 2.4 GHz	S21 to 3.4 GHz	S21 to 4.1 GHz
Anechoic Chamber	−22.002 dB	−30.002 dB	−34.226 dB	−45.105 dB
C_2_H_4_O_2_ 1%	−24.013 dB	−30.906 dB	−35.774 dB	−46.012 dB
C_2_H_4_O_2_ 3%	−24.746 dB	−30.776 dB	−36.014 dB	−47.565 dB
C_2_H_4_O_2_ 10%	−25.610 dB	−31.565 dB	−36.619 dB	−47.134 dB
C_2_H_4_O_2_ 20%	−26.001 dB	−32.103 dB	−37.245 dB	−48.166 dB
C_2_H_4_O_2_ 30%	−27.484 dB	−32.198 dB	−37.846 dB	−48.446 dB

## References

[1] O’Hara J.F., Singh R., Brener I., Smirnova E., Han J., Taylor A.J., Zhang W. (2008). Thin film sensing with planar terahertz metamaterials: Sensitivity and limitations. Opt. Express.

[2] Withayachumnankul W., Lin H., Serita K., Shah C.M., Sriram S., Bhaskaran M., Tonouchi M., Fumeaux C., Abbott D. (2012). Sub diffraction thin film sensing with planar terahertz metamaterials. Opt. Express.

[3] Zarifi M.H., Daneshmand M. (2016). Wide dynamic range microwave planar coupled ring resonator for sensing applications. Appl. Phys. Lett..

[4] Albishi A.M., Ramahi O.M. (2018). Highly Sensitive Microwaves Sensors for Fluid Concentration Measurements. IEEE Microw. Wirel. Compon. Lett..

[5] Zarifi M.H., Daneshmand M. (2017). High-resolution RFID liquid sensing using a chipless tag. IEEE Microw. Wirel. Compon. Lett..

[6] Ebrahimi A., Withayachumnankul W., Al-Sarawi S.F., Abbott D. Microwave microfluidic sensor based on microstrip-line-coupled complementary resonator. Proceedings of the 2016 IEEE 2nd Australian Microwave Symposium (AMS).

[7] Zarifi M.H., Daneshmand M. (2017). Monitoring Solid Particle Deposition in Lossy Medium Using Planar Resonator Sensor. IEEE Sens. J..

[8] Zarifi M.H., Sadabadi H., Hejazi S.H., Daneshmand M., Sanati-Nezhad A. (2018). Noncontact and Nonintrusive Microwave-Microfluidic Flow Sensor for Energy and Biomedical Engineering. Sci. Rep..

[9] Abdolrazzaghi M., Zarifi M.H., Daneshmand M. (2016). Wireless communication in feedback-assisted active sensors. IEEE Sens. J..

[10] Balanis C.A. (2005). Analysis and Design.

[11] Pozar D.M. (1992). Microstrip Antennas. Proc. IEEE.

[12] Veselago V.G. (1968). The electrodynamics of substance with simultaneously negative values of ε and μ. Sov. Phys. Uspekhi.

[13] Pendry J.B., Holden A.J., Robbins D.J., Stewart W.J. (1999). Magnetism from conductors and enhanced nonlinear phenomena. IEEE Trans. Microw. Theory Tech..

[14] Bai Q., Liu C., Chen J., Cheng C., Kang M. (2010). Tunable slow light in semiconductor metamaterial in a broad terahertz regime. J. Appl. Phys..

[15] Ishimaru A., Jaruwatanadilok S., Kuga Y. (2005). Generalized surface plasmon resonance srensors using metamaterials and negative index materials. Prog. Electromagn. Res..

[16] He S., Jin Y., Ruan Z.C., Kuang J.G. (2005). On subwavelength and open resonators involving metamaterials of negative refraction index. New J. Phys..

[17] Cong L., Tan S., Yahiaoui R., Yan F., Zhang W., Singh R. (2015). Experimental demonstration of ultrasensitive sensing with terahertz metamaterial absorbers: A comparison with the metasurfaces. Appl. Phys. Lett..

[18] Yahiaoui R., Strikwerda A.C., Jepsen P.U. (2016). Terahertz Plasmonic Structure with Enhanced Sensing Capabilities. IEEE Sens. J..

[19] Yahiaoui R., Tan S., Cong L., Singh R., Yan F., Zhang W. (2015). Multispectral terahertz sensing with highly flexible ultrathin metamaterial absorber. J. Appl. Phys..

[20] Alù A., Engheta N. (2008). Dielectric sensing in ε-near-zero narrow waveguide channels. Phys. Rev. B.

[21] Caloz C., Itoh T. (2005). Electromagnetic Metamaterial: Transmission Line Theory and Microwave Application.

[22] Kaschke J., Blume L., Wu L., Thiel M., Bade K., Yang Z., Wegener M. (2015). A helical metamaterial for broadband circular polarization conversion. Adv. Opt. Mater..

[23] Barreto E.L., de Mendonca L.M. A Novel Planar Fractal Antenna with CPW-Feed and Partial Ground Plane Removal for C-Band and S-Band Applications. Proceedings of the 2016 IEEE AP-S Symposium on Antennas and Propagation and USNC-URSI Radio Science Meeting.

[24] Campos A.L.P., d’Assuncao A.G., De Mendonca L.M. (2002). Scattering by FSS on anisotropic substrate for TE and TM excitation. IEEE Trans. Microw. Theory Tech..

[25] Li X.S., Xu K.D., Liu Z.M., Zhou D.Y., Du F. (2015). Metamaterial Extends Patch. Microw. RF.

[26] Li L.W., Li Y.N., Yeo T.S. (2010). A broadband and high-gain metamaterial microstrip antenna. Appl. Phys. Lett..

[27] Schreiber D., Gupta M., Cravey R. (2011). Comparative study of 1-D and 2-D metamaterial lens for microwave nondestructive evaluation of dielectric materials. Sens. Actuators A Phys..

[28] Howell J.Q. Microstrip antennas. Proceedings of the Antennas and Propagation Society International Symposium.

[29] Filho R.C., Araújo J.H., Ginani M.F., d’Assunção A.G., Martins R.A., d’Assunção A.G., Mendonça L.M. (2010). Simulation and measurement of inset-fed microstrip patch antennas on BiNbO substrates. Microw. Opt. Technol. Lett..

[30] Boybay M.S., Ramahi O.M. (2012). Material Characterization using complementary split-ring resonators. IEEE Trans. Instrum. Meas..

[31] Lee C.S., Yang C.L. (2014). Complementary split-ring resonators for measuring dielectric constants and loss tagents. IEEE Microw. Wirel. Compon. Lett..

[32] Yang C.L., Lee C.S., Chen K.W., Chen K.Z. (2016). Noncotact measurement of complex permittivity and thickness bay using planar resonators. IEEE Trans. Microw. Theory Tech..

[33] Sousa Neto M.P., Fernandes H.C.C. (2014). Um Estudo de Metamaterial em Antenas de Microfita. Ph.D. Thesis.

[34] Weiland T., Schumann R., Greegor R.B., Parazzoli C.G., Vetter A.M., Smith D.R., Vier D.C., Schultz S. (2001). Ab Initio Numerical Simulation of Left-handed Metamaterials: Comparison of Calculations and experiments. J. Appl. Phys..

[35] Rahman N.A., Zakaria Z., Rahim R.A., Dasril Y., Bahar A.A.M., Azuan A. (2017). Planar Microwave Sensors for Accurate Measurement of Material Characterization: A Review. TELKOMNIKA.

[36] Salim A., Lim S. (2016). Complementary Split-Ring Resonator-Loaded Microfluidic Ethanol Chemical Sensor. Sensors.

[37] Withayachumnankul W., Jaruwongrungsee K., Tuantranont A., Fumeaux C., Abbott D. (2013). Metamaterial-based microfluidic sensor for dielectric characterization. Sens. Actuators A Phys..

